# Monocyte subtype counts are associated with 10-year cardiovascular disease risk as determined by the Framingham Risk Score among subjects of the LIFE-Adult study

**DOI:** 10.1371/journal.pone.0247480

**Published:** 2021-03-01

**Authors:** Samira Zeynalova, Karolin Bucksch, Markus Scholz, Maryam Yahiaoui-Doktor, Melanie Gross, Markus Löffler, Susanne Melzer, Attila Tárnok

**Affiliations:** 1 LIFE–Leipzig Research Center for Civilization Diseases, Universität Leipzig, Leipzig, Germany; 2 Institute for Medical Informatics, Statistics and Epidemiology, Universität Leipzig, Leipzig, Germany; 3 Klinik für Kinder- und Jugendmedizin, DRK-Krankenhaus Chemnitz-Rabenstein, Chemnitz, Germany; 4 Zentrum für Klinische Studien, ZKS Leipzig, Universität Leipzig, Leipzig, Germany; 5 Department for Therapy Validation, Fraunhofer Institute for Cell Therapy and Immunology IZI, Leipzig, Germany; Universidade de Sao Paulo Instituto de Quimica, BRAZIL

## Abstract

Coronary heart disease, an inflammatory disease, is the leading cause of death globally. White blood cell counts (including monocytes) are easily available biomarkers of systemic inflammation. Monocyte subtypes can be measured by flow cytometry and classified into classical (CD14^high^, CD16^neg^), intermediate (CD14^high^, CD16^+^) and non-classical (CD14^+^, CD16^high^) with distinct functional properties. The goal of this study was to investigate the association of monocyte total count and its subtypes with cardiovascular risk groups defined by the Framingham Risk Score, which is used to estimate the 10-year risk of developing myocardial infarction or predict mortality following coronary heart disease. We also aimed to investigate whether monocyte counts are associated with relevant cardiovascular risk factors not included in the Framingham Risk Score, such as carotid atherosclerotic plaque and intima-media thickness. Our data came from the LIFE-Adult study, a population-based cohort study of 10,000 randomly selected participants in Leipzig, Germany. Data was gathered using self-administered questionnaires and physical examinations. Carotid plaques and intima-media thickness were measured using carotid artery sonography. Monocyte subtypes in blood were determined by 10-color flow cytometry for a total of 690 individuals. In a multivariate regression analysis adjusting for the risk factors BMI, intima-media thickness, presence of carotid plaques and diabetes mellitus, monocyte subtypes and total count were found to be significantly associated with the dichotomized Framingham Risk Score (≥10% versus <10%): Odds ratios [95% confidence interval] for monocyte subtypes: classical: 11.19 [3.79–34.26]; intermediate: 2.27 [1.11–4.71]; non-classical: 4.18 [1.75–10.20]; total: 14.59 [4.61–47.95]. In absence of prospective data, the FRS was used as a surrogate for CHD. Our results indicate that monocyte counts could provide useful predictive value for cardiovascular disease risk.

## Introduction

The prevalence of coronary heart disease (CHD) is expected to constantly increase in the whole world [1] within the next decade due to high exposure to life-style risk factors (e.g. a sedentary life style, hypercaloric diets, tobacco smoking as well as alcohol consumption) and increasing life expectancy. Many risk factors predisposing individuals to CHD are described in the literature [2–4]. In 1959, Dawber et al. [5] identified a number of CHD risk factors and developed the Framingham Risk Score (FRS) for the US population, enabling clinicians to estimate an individual’s risk of developing CHD within the next 10 years.

CHD is an inflammatory disease, and one of the simplest biomarkers to measure systemic inflammation is the white blood cell count [6]. It is still unclear whether immune cell subtypes and especially which subtypes might predict cardiovascular events [7,8]. However, as all monocyte subtypes are involved in the immune system response and inflammatory processes [9], we hypothesized that these cells are promising biomarkers for CHD risk prediction.

Using multi-color flow cytometry, human peripheral blood monocytes are phenotypically grouped into classical (CD14^high^CD16^neg^), non-classical (CD14^dim^CD16^high^) and intermediate (CD14^high^CD16^pos^) subtypes [10]. These subtypes have distinct functional properties: classical monocytes are highly phagocytic, whereas non-classical monocytes are pro-inflammatory and stimulate T-cells.

Therefore, we aimed to investigate whether monocyte subtypes counts are associated with cardiovascular risk groups according to the FRS. We also aimed to investigate whether monocyte counts are independent risk factors for CHD after adjusting for parameters not included in the FRS calculation, such as BMI, carotid atherosclerotic plaque, intima-media thickness and diabetes. Type 2 diabetes has been found to be associated with increased cardiovascular morbidity [11–13].

## Materials and methods

### Subjects

The LIFE-Adult study is a population-based cohort study of 10,000 randomly selected inhabitants of Leipzig, a city with over 600,000 inhabitants in the east of Germany. The study covers adults between the ages of 18 and 79 years with particular deep phenotyping in participants aged 60 and older [14]. Baseline enrolment was conducted from August 2011 to November 2014 by the Leipzig Research Centre for Civilization Diseases (LIFE).

The study was approved by the responsible institutional ethics board of the Medical Faculty of the University of Leipzig (Vote of the ethics committee No 263-2009-14122009). All participants gave their written informed consent at study inclusion.

### Blood sample collection, preparation and staining (flow cytometry)

**Fig 1:**
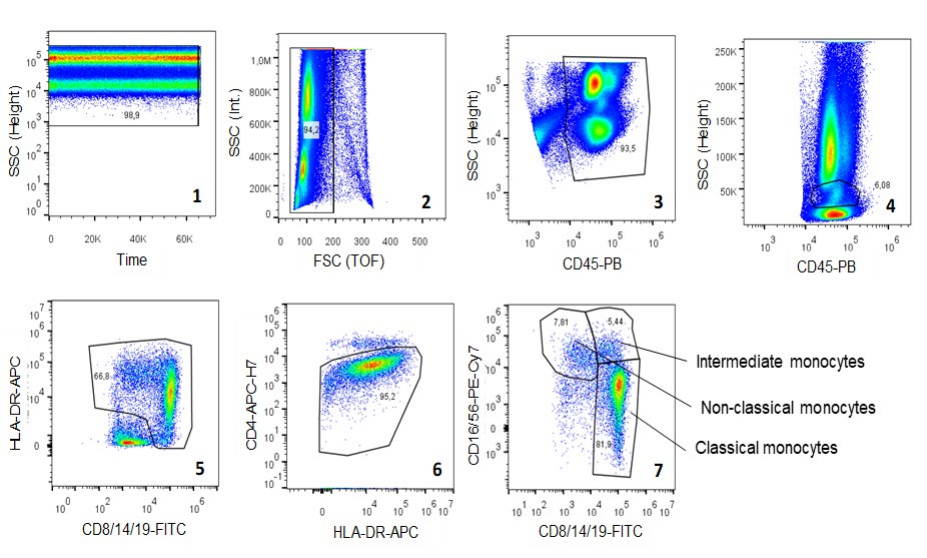
Gating of the monocyte subtypes. Exclusion of air bubbles (plot 1), doublets (plot 2), CD45neg cells (plot 3), lymphocytes and granulocytes (plot 4), HLA-DR^neg^ cells (plot 5) and CD4^high^ cells (plot 6) in a first step with subsequent distinction of three monocyte subtypes based on the expression of CD14 and CD16 (plot 7). Abbreviations: SSC—sideward scatter, FSC—forward scatter, TOF—time of flight, PB—Pacific Blue, FITC—fluorescein isothiocyanate, APC–allophycocyanin, H7—Highlight 750, PE–phycoerythrin, Cy–cyanines, CD—cluster of differentiation protein, HLA-DR—human leucocyte antigen-antigen D related.

Peripheral blood samples were prepared and processed for flow cytometric analysis and analyzed by flow cytometry according to the established LIFE Cytomics protocol, the details of which are found elsewhere [15]. Manual gating was performed using FlowJo V.10.0.7 (Tree Star, Asland, OR). A liberal gating strategy with overlapping gates was used in sequential steps (see Fig 1): exclusion of air bubbles (1: time versus sideward scatter (SSC) plot), duplicates (2: FSC-TOF versus forward scatter (FSC) plot) and CD45^neg^ events (3: CD45 versus SSC plot); Gating of monocytes (4: CD45 versus SSC plot); exclusion of contaminating lymphocytes and granulocytes by exclusion of HLA-DR^neg^ (5: CD8/14/19 versus HLA-DR plot) and CD4^high^ events (6: HLA-DR versus CD4 plot); and finally (7) the distinction of classical (CD14^high^, CD16^neg^), intermediate (CD14^high^, CD16^pos^) and non-classical monocytes (CD14^dim^, CD16^high^). For setting the gate borders between the monocyte subtypes, we referred to the work of Zawada et al. [7] and used rectangular gating strategies. Event counts were used to calculate relative frequencies of cells. In order to obtain absolute cell numbers, the cell numbers per unit volume of blood (10^9^ L^-1^) were calculated using total WBC.

### Carotid ultrasound

Atherosclerotic plaques and intima media thickness of the arterial wall were assessed by ultrasound as described elsewhere [14]. Plaques were defined to be present where at least one of the eight measurement points (arteria carotis communis, arteria carotis interna, arteria carotis externa and bulbus on the right and left side) had a deposit of ≥1.5 mm. Intima-media thickness was measured in mm for right and left sides. Since both are highly correlated (Pearson correlation coefficient of 0.7), its mean value was calculated and included in the analysis.

### Framingham Risk Score

The Framingham Risk Score is a scoring system used to assess a sex-specific individual risk in people aged 18 to 80 without known CHD of developing cardiovascular disease events, i.e. myocardial infarction in the next 10 years. The FRS uses the risk factors: age, sex, smoking status (non-smoker, current smoker), value of systolic blood pressure, total cholesterol and high-density lipoprotein cholesterol. We grouped individuals in three FRS categories as described by Bosomworth [16]: "low risk" is assumed if the score is less than 10%, "moderate" if it is 10–20% and "high" if the score is 20% or higher.

### Statistical analysis

Patient demographic characteristics were presented as median (first and third quartile) values for continuous variables and frequency (percentage) for categorical variables. Comparisons between the three FRS groups were made using the Kruskal-Wallis test (as a global test) or the Mann-Whitney U test (as a two-group test) for continuous variables, and Chi-squared test for categorical variables. The association between FRS and the explanatory variables BMI, intima-media thickness, presence of carotid plaques, the self-reported medical history data for diabetes mellitus and the monocyte counts were assessed by linear regression analyses for continuous FRS and by logistic regression for the comparison of low risk group (<10%) and moderate or high risk group (≥10%). Since the FRS is calculated based on age, sex, smoking status, systolic blood pressure, total cholesterol and high-density lipoprotein cholesterol, we did not adjust for these as variables in the regression analyses. Due to a low sample size and as the demographic characteristics were similar between the groups, we joined the FRS moderate and high-risk groups together in the logistic regression analysis. Regression models were calculated individually for each monocyte subtype (classical, intermediate, non-classical subtypes and total). Counts of monocyte subtype were not normally distributed and therefore log10 transformed. Means and 95% confidence intervals of monocyte subtypes were estimated depending on the three different FRS groups.

All reported p-values were two-sided, and p-values lower than 0.05 were considered as statistically significant. For multiple comparisons, p-values were Bonferroni-corrected (by multiplying the original p-value with the number of tested null hypotheses: 14 global tests, 13 two-sample tests). Statistical analyses were carried out with R 3.6.1 for Windows (R Core Team. R: A language and environment for statistical computing. R Foundation for Statistical Computing, Vienna, Austria. URL: https://www.R-project.org).

## Results

Blood samples of 871 LIFE-Adult subjects were analyzed by 10-color flow cytometry as described by Bocsi and colleagues [17]. Patients with self-reported previous myocardial infarction, bypass grafting or stroke were excluded from further analysis. In total, 690 individuals (309 males, 381 females) were eligible for statistical analyses. As shown in Table 1, there were 462 individuals (134 males, 328 females) in the FRS low risk group, 171 individuals (124 males, 47 females) in the intermediate risk group and 57 individuals (51 males, 6 females) in the high risk group. Men were at higher risk than women. The medians of age, systolic blood pressure and BMI, as well as the relative number of men were statistically significantly higher with increasing FRS. Statistically significant differences between the low and moderate score groups were detected for smoking status, as well as for intima-media thickness, the classical, non-classical and total monocyte subtypes, the frequency of carotid plaques and diabetes mellitus, which were all higher in the moderate group. Only for total cholesterol, the differences in the medians between the risk groups were not statistically significant. In contrast, the median of high-density lipoprotein cholesterol was significantly lower.

**Table 1:** Characteristics of 690 individuals from LIFE-Adult with Cytomics assessment and grouped by calculated Framingham Risk Score: Low (<10%), moderate (10%—<20%) and high risk (≥20%) groups.

	Framingham Risk Score (in %)			group differences		
Characteristics	<10	10-<20	≥20	global	<10 vs. 10 - <20	10 - <20 vs. ≥20
	(n = 462)	(n = 171)	(n = 57)	p-value^a^	p-value^b^	p-value^b^
Age, median (IQR), years	51.6 (43.4–63.3)	66.3 (57.2–71.2)	69.6 (65.3–74.7)	<0.001	<0.001	0.023
Female, n (%)	328 (71.0)	47 (27.5)	6 (10.5)	<0.001	<0.001	0.189
Smoking status, n (%)				<0.001	<0.001	≈1
Never smoker	303 (65.6)	68 (39.8)	24 (42.1)			
Former smoker	103 (22.3)	64 (37.4)	18 (31.6)			
Current smoker	56 (12.1)	39 (22.8)	15 (26.3)			
Systolic BP, median (IQR), mmHg	123.0 (115.0–132.4)	136.5 (124.2–149.5)	147.0 (134.5–161.0)	<0.001	<0.001	0.004
Total cholesterol, median (IQR), mmol/L	5.57 (4.82–6.23)	5.86 (5.08–6.66)	5.72 (4.99–6.34)	0.066		
HDL cholesterol, median (IQR), mmol/L	1.69 (1.41–2.02)	1.53 (1.26–1.80)	1.11 (0.99–1.24)	<0.001	<0.001	<0.001
BMI, median (IQR), kg/m^2^	25.5 (23.2–28.8)	27.3 (24.8–30.3)	29.4 (27.1–32.3)	<0.001	<0.001	0.010
Intima-media thickness^c^, median (IQR), mm	0.68 (0.58–0.78)	0.82 (0.72–0.91)	0.82 (0.74–0.94)	<0.001	<0.001	≈1
Present carotid plaques, n (%)	95 (20.6)	93 (54.4)	40 (70.2)	<0.001	<0.001	0.683
Present diabetes mellitus^d^, n (%)	21 (4.6)	31 (18.3)	12 (21.1)	<0.001	<0.001	≈1
Classical monocyte count, median (IQR) [min, max], 10^9^ cells/L	0.233 (0.176–0.311) [0.051, 0.761]	0.271 (0.209–0.353) [0.072, 0.909]	0.304 (0.201–0.374) [0.086, 0.631]	<0.001	0.001	≈1
Intermediate monocyte count, median (IQR) [min, max], 10^9^ cells/L	0.016 (0.011–0.022) [0.001, 0.089]	0.019 (0.012–0.025) [0.003, 0.120]	0.020 (0.013–0.029) [0.003, 0.053]	0.022	0.059	≈1
Non-classical monocyte count, median (IQR) [min, max], 10^9^ cells/L	0.028 (0.020–0.038) [0.005, 0.122]	0.033 (0.025–0.045) [0.008, 0.125]	0.042 (0.026–0.060) [0.003, 0.120]	<0.001	<0.001	≈1
Total monocyte count, median (IQR) [min, max], 10^9^ cells/L	0.299 (0.226–0.384) [0.079, 0.900]	0.345 (0.276–0.442) [0.108, 0.995]	0.374 (0.261–0.462) [0.129, 0.783]	<0.001	<0.001	≈1

IQR: interquartile range.

^a^p-values of global test: p-values are Bonferroni-corrected for multiple comparisons; Kruskal-Wallis test for continuous variables; Chi-squared test for categorical variables.

^b^p-values of two-sample tests: only if Bonferroni-corrected p-value of global test < 0.05; p-values of two-sample tests are Bonferroni-corrected for multiple comparisons (each column separately); Mann-Whitney U test for continuous variables; Chi-squared test for categorical variables.

^c^Intima-media thickness: 28 missing observations (Framingham Risk Score (in %) <10/10-<20/≥20: 20/4/4).

^d^Present Diabetes mellitus: 3 missing observations (Framingham Risk Score (in %) <10/10-<20/≥20: 1/2/0).

**Fig 2:**
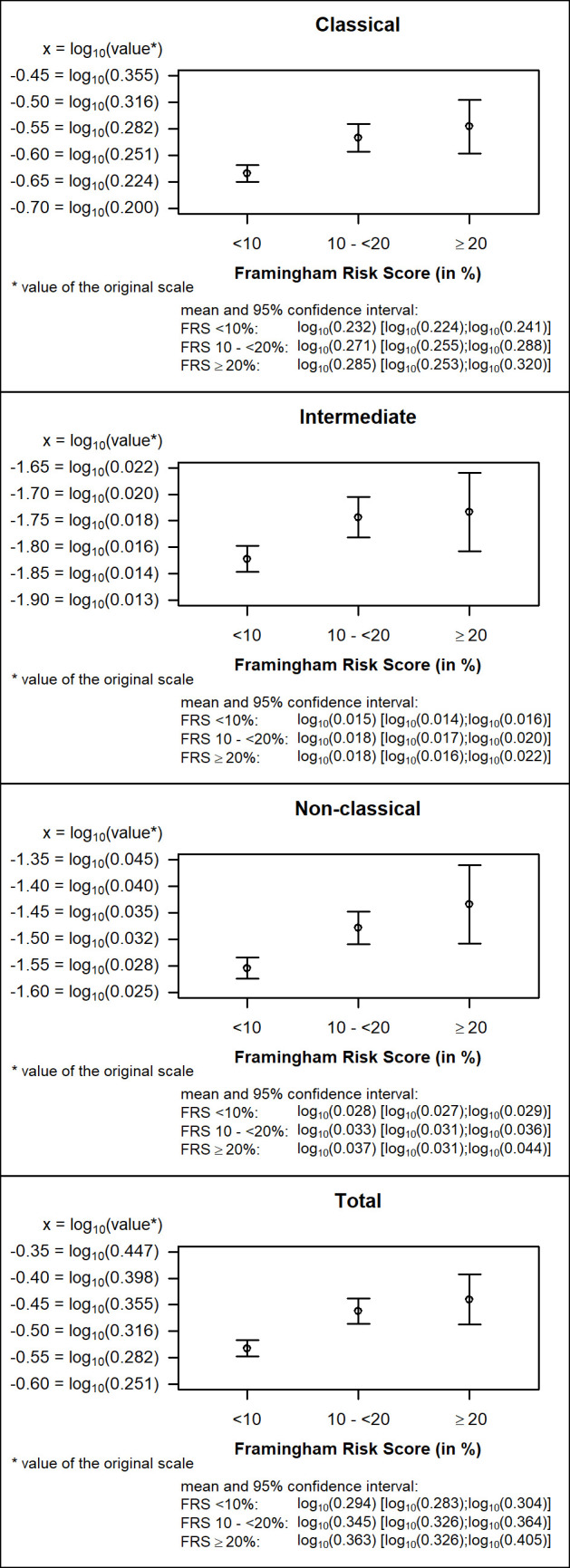
Comparison of Framingham risk estimates. Mean and 95% confidence interval of logarithmized classical, intermediate, non-classical and total monocyte count (10^9^ cells/L).

In the univariate analyses, as shown in Fig 2, for all monocyte subtypes, the Framingham Risk Score group <10% (low risk) had the lowest counts. We saw a continuous increase for higher FRS groups.

In multivariate linear regression analyses performed for all monocyte subtypes separately and adjusting for BMI, intima-media thickness, presence of carotid plaques and diabetes mellitus, all these variables and the specific logarithmized monocyte count were found to be significantly associated with FRS (see Table 2).

**Table 2:** Linear regression analyses for the different monocyte subtypes (classical, intermediate, non-classical and total).

		coefficient	95% confidence interval	standardized coefficient	p-value
**Classical**	BMI	0.262	0.159–0.365	0.170	<0.001
	Intima-media thickness	15.315	12.043–18.588	0.338	<0.001
	Present carotid plaques	2.884	1.815–3.953	0.194	<0.001
	Present diabetes mellitus	1.976	0.340–3.612	0.081	0.018
	Logarithmic monocyte count	5.914	3.473–8.355	0.152	<0.001
**Intermediate**	BMI	0.258	0.153–0.362	0.167	<0.001
	Intima-media thickness	14.813	11.490–18.137	0.327	<0.001
	Present carotid plaques	2.990	1.910–4.070	0.201	<0.001
	Present diabetes mellitus	2.144	0.492–3.796	0.088	0.011
	Logarithmic monocyte count	2.346	0.683–4.008	0.090	0.006
**Non-classical**	BMI	0.241	0.136–0.345	0.156	<0.001
	Intima-media thickness	14.468	11.158–17.778	0.319	<0.001
	Present carotid plaques	3.099	2.025–4.174	0.209	<0.001
	Present diabetes mellitus	1.935	0.288–3.582	0.079	0.021
	Logarithmic monocyte count	4.157	2.130–6.185	0.132	<0.001
**Total**	BMI	0.257	0.154–0.359	0.166	<0.001
	Intima-media thickness	15.049	11.784–18.314	0.332	<0.001
	Present carotid plaques	2.917	1.851–3.983	0.196	<0.001
	Present diabetes mellitus	1.915	0.282–3.548	0.079	0.022
	Logarithmic monocyte count	6.749	4.156–9.341	0.164	<0.001

659 valid observations (31 deleted due to missingness). Dependent variable: Framingham Risk Score (continuous). Independent variables: BMI (continuous, kg/m^2^), Intima-media thickness (continuous, mm), Present carotid plaques (not present (reference) versus present), Present diabetes mellitus (not present (reference) versus present), Logarithmic monocyte count (continuous, 10^9^ cells/L).

**Fig 3:**
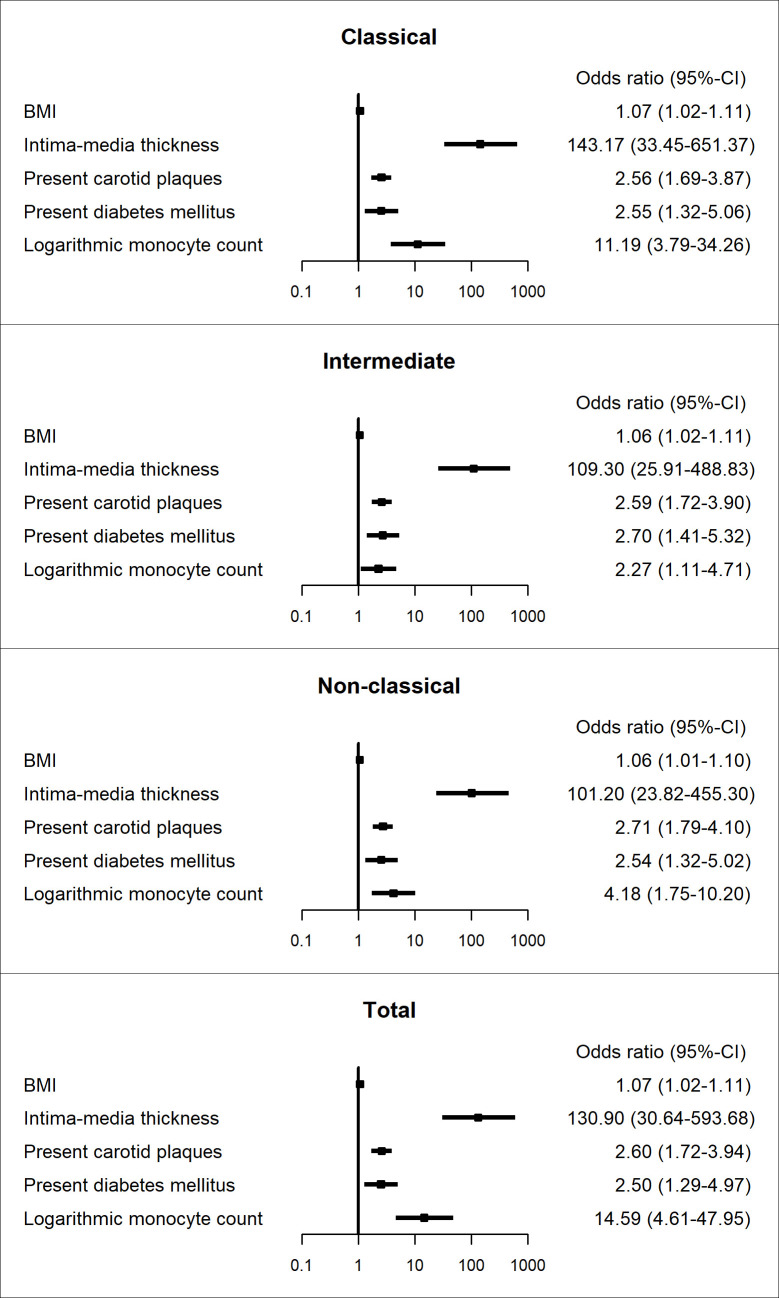
Odds ratios of binary logistic regression model. Dependent variable: Framingham Risk Score (≥10% versus <10%). Displayed separately for the different monocyte subtypes. 659 valid observations (31 deleted due to missingness). Independent variables: BMI (continuous, kg/m^2^), Intima-media thickness (continuous, mm), Present carotid plaques (not present (reference) versus present), Present diabetes mellitus (not present (reference) versus present), Logarithmic monocyte count (continuous, 10^9^ cells/L).

This was confirmed by logistic regression analysis of the dichotomized risk score (see Fig 3). The risks of increased FRS were higher with increased logarithmic monocyte count for all monocyte subtypes and the total count (Odds ratios [95% confidence interval] for monocyte subtypes: classical: 11.19 [3.79–34.26]; intermediate: 2.27 [1.11–4.71]; non-classical: 4.18 [1.75–10.20]; total: 14.59 [4.61–47.95]). Additionally, higher intima-media thickness was also associated with higher FRS, as well as present carotid plaques, present diabetes mellitus and higher BMI.

## Discussion

In the present study, we analyzed the potential value of cytometry-derived monocyte subtypes for improved cardiovascular risk evaluation by contrasting them with FRS categories.

State of the art: Madjid and Fatemi summarized in their review article the multiple functions of leukocytes in the initiation and progression of CHD [18]. Leukocytes can induce endothelial and myocardial damage by releasing proteases and peroxidases, accelerating also plaque instability, plug coronary vessels (decreased perfusion) and initiate abnormal WBC aggregation. Waterhouse and colleagues showed in 2008 that monocytes have the highest predictive potential regarding cardiovascular outcome of symptomatic CHD patients [19]. Lo and Colleagues studied correlations of intermediate monocyte count and cardiovascular risk. They found that intermediate monocyte count could predict severe CAD [20]. Increased counts for non-classical and intermediate monocytes were reported in a cohort of dialysis patients compared to healthy controls contributing to an accelerated atherogenesis in patients with impaired renal function [21].

Findings of the present study: We focused on monocytes and their subtypes and neglected other leukocyte subtypes. Compared to other studies considering a specific disease population, we considered a population-based sample of randomly selected participants. It seemed interesting to us to assess the risk of cardiovascular diseases for this cohort in particular.

We found that monocyte subtypes were significantly associated with FRS. In particular, based on available literature, we expected that the intermediate subtype might have a strong association with cardiovascular risk. Our analyses suggest that our innovative monocyte subtype differentiation (including the analysis of the intermediate subset) has the potential to improve cardiovascular risk prediction.

Differences between participants with a moderate FRS (10-<20%) and a high FRS (≥20%) regarding monocyte count subtypes were not statistically significant. We suspect that a reason might be the subjectivity of this 20% cut point. Concerning the monocyte count, it does not seem to be necessary to form three FRS categories, as significant differences can already be seen when looking at the low-risk group compared to the individuals with an FRS ≥10%. The relatively low number of cases in the high-risk group (n = 57) compared to n = 462 in the low-risk group and n = 171 in the moderate-risk group might also be a reason why no statistical significance could be found.

In our population, 99% of the participants were European. The validity of the current FRS to predict the individual probability of CHD progression for European cohorts has previously been questioned [22–24]. We believe that the FRS could accordingly be modified or extended in the near future, perhaps using follow-up data of the LIFE-Adult study. Based on our current findings, we think that monocyte subtypes have the potential to improve CHD risk prediction in symptom free individuals.

Limitations of methods: When we performed our cytometric analysis, the nature of the intermediate monocyte subtype was not clearly defined. As no specific markers were available, we were limited to the subtype differentiation based on the CD14 and CD16 expressions. Besides the limitations of manual vs. automated gating techniques, monocyte subtype differentiation is especially difficult in the absence of subtype-specific markers. The limitations in subtype differentiation by manual gating in this study might be compensated by the large data set (nearly 700 individual measurements). As the development of specific monocyte subtype markers is still ongoing and no specific, intermediate monocyte marker was commercially available yet, we used the best method available at the time. However, new markers and marker combinations for further characterizing and sub-dividing monocytes may reveal new associations (not available for this analysis).

Outlook: Longitudinal studies are necessary to evaluate our hypothesis about the association of monocyte subtypes and total monocyte count with cardiovascular disease, especially the occurrence of myocardial infarction in the further course. In LIFE-Adult for instance, follow-up data is currently being collected, which might aid in identifying risk prediction factors and developing models to identify individuals at high risk for CHD and to predict future events in patients with CHD. These factors could indicate which patients would most likely benefit from prevention, such as preventive drugs or a change of lifestyle. Future research is needed to find out if changes in monocyte counts can not only predict CHD risk, but might also serve as a prevention or therapy target of cardiovascular disease.

## Conclusions

We could show that monocyte subtypes are associated with risk groups of CHD. Therefore, these subtypes are a potential biomarker for risk prediction. Predictive potential needs to be assessed in a longitudinal study.

## Supporting information

S1 Fig Relationship between intima-media thickness and monocyte count depending on Framingham Risk Score categories. (TIF)

S1 Appendix Regression analyses. (PDF)
