# Supplementary figures and images for: Monocyte subtype counts are associated with 10-year cardiovascular disease risk as determined by the Framingham Risk Score among subjects of the LIFE-Adult study
Source: PLoS One. 2021 Mar 1;16(3):e0247480. doi: 10.1371/journal.pone.0247480 (PMC7920341; doi:10.1371/journal.pone.0247480)

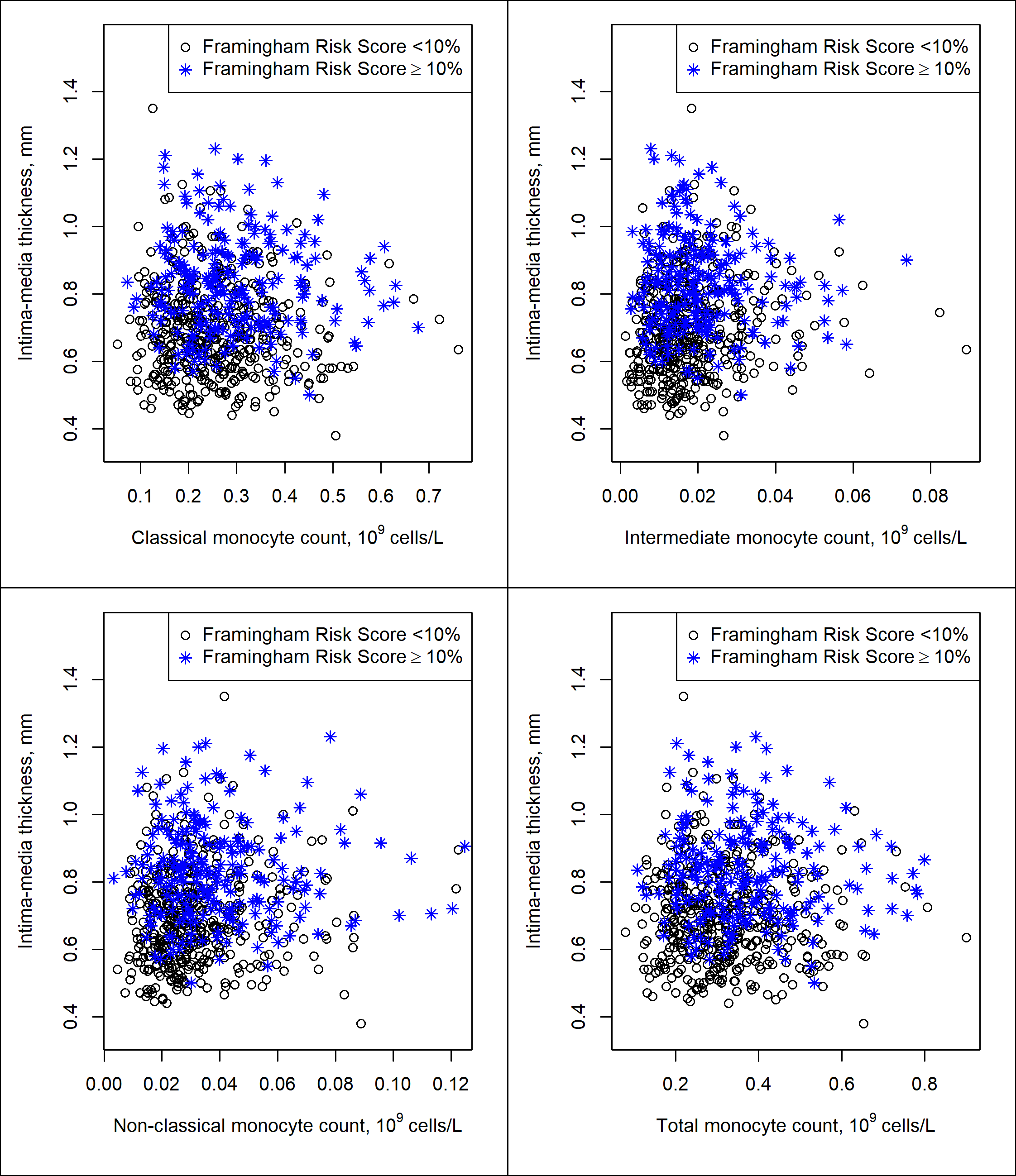

Supplement: S1 Fig — (TIF) [file pone.0247480.s001.tif]
