# Supplementary material for: Monocyte subtype counts are associated with 10-year cardiovascular disease risk as determined by the Framingham Risk Score among subjects of the LIFE-Adult study
Source: PLoS One. 2021 Mar 1;16(3):e0247480. doi: 10.1371/journal.pone.0247480 (PMC7920341; doi:10.1371/journal.pone.0247480)
Supplement: S1 Appendix — (PDF) [file pone.0247480.s002.pdf]

APPENDIX: Zeynalova S, Bucksch K, Scholz M, et al.: Monocyte subtype counts are associated with 10-year cardiovascular disease risk as determined by the Framingham Risk Score among subjects of the LIFE-Adult study

S1 Appendix: Regression analyses

A) Linear regression analyses for the different monocyte subtypes (classical, intermediate, non-classical and total).

Dependent variable: Framingham Risk Score (continuous).

Independent variables: BMI (continuous, kg/m²), Intima-media thickness (continuous, mm), Present carotid plaques (not present (reference) versus present), Present diabetes mellitus (not present (reference) versus present), Logarithmic monocyte count (continuous, 109 cells/L).

659 valid observations (31 deleted due to missingness).

Calculated with R 3.6.1 for Windows. Using functions lm, summary.lm and confint of package stats.

Goodness of fit: R-squared and adjusted R-squared were calculated.

Model 1: Monocyte subtype "Classical":

	coefficient	standard error	p-value	confidence interval
Intercept	-8.881	1.959	6.91E-06	(-12.728)-(-5.034)
BMI	0.262	0.052	7.34E-07	0.159-0.365
Intima-media thickness	15.315	1.667	<2E-16	12.043-18.588
Present carotid plaques	2.884	0.544	1.60E-07	1.815-3.953
Present diabetes mellitus	1.976	0.833	1.80E-02	0.340-3.612
Logarithmic monocyte count	5.914	1.243	2.42E-06	3.473-8.355

Goodness of fit:

R-squared	Adjusted R-squared
0.339	0.334

Model 2: Monocyte subtype "Intermediate":

	coefficient	standard error	p-value	confidence interval
Intercept	-7.825	2.510	1.90E-03	(-12.753)-(-2.896)
BMI	0.258	0.053	1.58E-06	0.153-0.362
Intima-media thickness	14.813	1.693	<2E-16	11.490-18.137
Present carotid plaques	2.990	0.550	7.70E-08	1.910-4.070
Present diabetes mellitus	2.144	0.841	1.11E-02	0.492-3.796
Logarithmic monocyte count	2.346	0.847	5.77E-03	0.683-4.008

Goodness of fit:

R-squared	Adjusted R-squared
0.324	0.319

Model 3: Monocyte subtype "Non-classical":

	coefficient	standard error	p-value	confidence interval
Intercept	-4.994	2.616	5.67E-02	(-10.131)-(0.143)
BMI	0.241	0.053	7.02E-06	0.136-0.345
Intima-media thickness	14.468	1.686	<2E-16	11.158-17.778
Present carotid plaques	3.099	0.547	2.19E-08	2.025-4.174
Present diabetes mellitus	1.935	0.839	2.14E-02	0.288-3.582
Logarithmic monocyte count	4.157	1.033	6.33E-05	2.130-6.185

Goodness of fit:

R-squared	Adjusted R-squared
0.333	0.328

Model 4: Total monocyte count:

	coefficient	standard error	p-value	confidence interval
Intercept	-8.721	1.946	8.73E-06	(-12.541)-(-4.900)
BMI	0.257	0.052	1.21E-06	0.154-0.359
Intima-media thickness	15.049	1.663	<2E-16	11.784-18.314
Present carotid plaques	2.917	0.543	1.06E-07	1.851-3.983
Present diabetes mellitus	1.915	0.832	2.16E-02	0.282-3.548
Logarithmic monocyte count	6.749	1.320	4.19E-07	4.156-9.341

Goodness of fit:

R-squared	Adjusted R-squared
0.343	0.338

B) Binary logistic regression analyses for the different monocyte subtypes (classical, intermediate, non-classical and total).

Dependent variable: Framingham Risk Score (≥10% versus <10%).

Independent variables: BMI (continuous, kg/m²), Intima-media thickness (continuous, mm), Present carotid plaques (not present (reference) versus present), Present diabetes mellitus (not present (reference) versus present), Logarithmic monocyte count (continuous, 109 cells/L).

659 valid observations (31 deleted due to missingness).

Calculated with R 3.6.1 for Windows. Using functions glm (family: binomial, link function: logit), summary.glm and confint of package stats.

Goodness of fit: The le Cessie-van Houwelingen-Copas-Hosmer unweighted sum of squares test for global goodness of fit was calculated using functions lrm and residuals.lrm of package rms.

Model 1: Monocyte subtype "Classical":

	coefficient	standard error	p-value	confidence interval
Intercept	-5.253	0.892	3.87E-09	(-7.034)-(-3.532)
BMI	0.065	0.022	3.67E-03	0.021-0.109
Intima-media thickness	4.964	0.756	5.23E-11	3.510-6.479
Present carotid plaques	0.939	0.211	8.24E-06	0.527-1.353
Present diabetes mellitus	0.935	0.342	6.26E-03	0.275-1.622
Logarithmic monocyte count	2.415	0.561	1.65E-05	1.332-3.534

Goodness of fit:

Sum of squared errors	Expected value|H0	SD	Z	p-value
107.945	106.307	0.689	2.377	0.017

Model 2: Monocyte subtype "Intermediate":

	coefficient	standard error	p-value	confidence interval
Intercept	-4.893	1.087	6.81E-06	(-7.054)-(-2.784)
BMI	0.059	0.022	6.92E-03	0.016-0.102
Intima-media thickness	4.694	0.748	3.52E-10	3.255-6.192
Present carotid plaques	0.951	0.208	4.29E-06	0.543-1.360
Present diabetes mellitus	0.992	0.338	3.28E-03	0.342-1.671
Logarithmic monocyte count	0.818	0.369	2.65E-02	0.103-1.550

Goodness of fit:

Sum of squared errors	Expected value|H0	SD	Z	p-value
110.402	109.078	0.623	2.123	0.034

Model 3: Monocyte subtype "Non-classical":

	coefficient	standard error	p-value	confidence interval
Intercept	-4.018	1.125	3.56E-04	(-6.248)-(-1.830)
BMI	0.055	0.022	1.34E-02	0.011-0.098
Intima-media thickness	4.617	0.751	8.05E-10	3.171-6.121
Present carotid plaques	0.997	0.211	2.22E-06	0.585-1.411
Present diabetes mellitus	0.933	0.339	6.00E-03	0.278-1.614
Logarithmic monocyte count	1.430	0.449	1.46E-03	0.558-2.322

Goodness of fit:

Sum of squared errors	Expected value|H0	SD	Z	p-value
109.200	108.004	0.650	1.840	0.066

Model 4: Total monocyte count:

	coefficient	standard error	p-value	confidence interval
Intercept	-5.264	0.890	3.28E-09	(-7.040)-(-3.548)
BMI	0.063	0.022	4.73E-03	0.019-0.107
Intima-media thickness	4.874	0.755	1.07E-10	3.422-6.386
Present carotid plaques	0.955	0.211	6.14E-06	0.541-1.370
Present diabetes mellitus	0.917	0.342	7.37E-03	0.256-1.604
Logarithmic monocyte count	2.680	0.596	6.91E-06	1.529-3.870

Goodness of fit:

Sum of squared errors	Expected value|H0	SD	Z	p-value
107.615	105.961	0.696	2.375	0.018
